# Supplementary material for: Elovanoid-N32 or RvD6-isomer decrease ACE2 and binding of S protein RBD after injury or INFγ in the eye
Source: Res Sq. 2020 Aug 11:rs.3.rs-55764. Preprint. [Version 1] doi: 10.21203/rs.3.rs-55764/v1 (PMC7430585; doi:10.21203/rs.3.rs-55764/v1)
Supplement: Supplement [file SupplementalFigures.pdf]

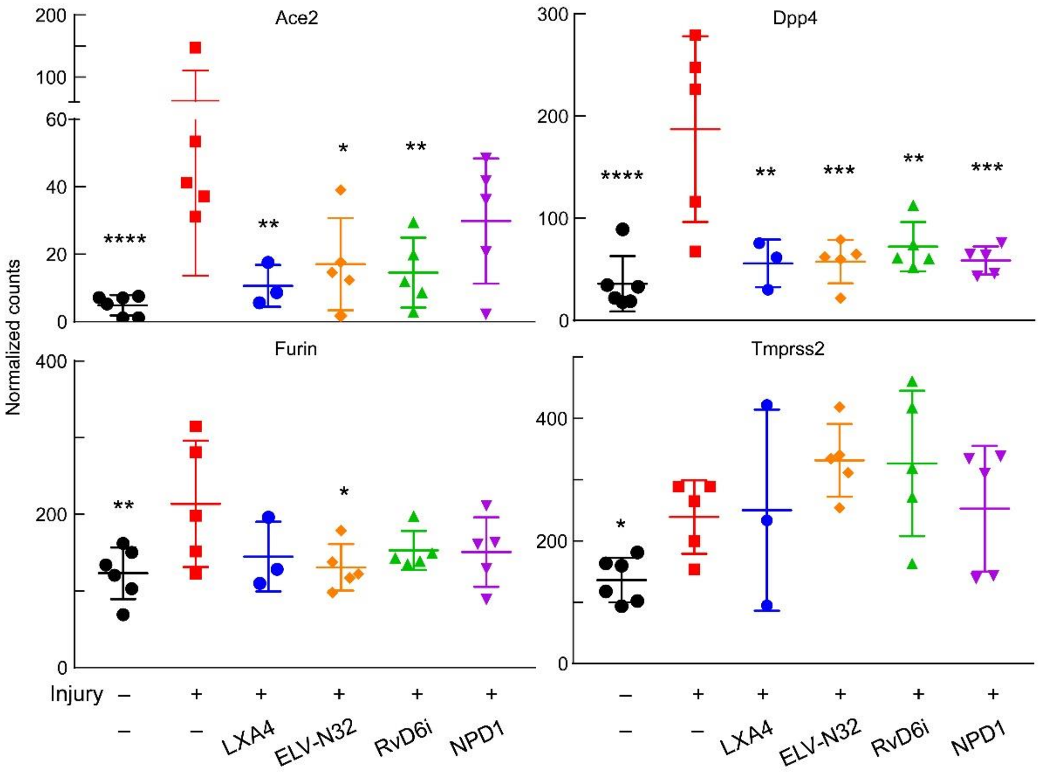

Supplementary Figure 1. Lipid mediators on gene expression of *Ace2*, *Dpp4*, *Furin*, and *Tmprss2* after cornea injury. RNA-seq data normalized counts, (mean and SD) analyzed by ANOVA-post hoc Dunnett's multiple comparisons test with vehicle as reference. *, $p < 0.05$, **, $p < 0.01$, ***, $p < 0.001$, and ****, $p < 0.0001$.

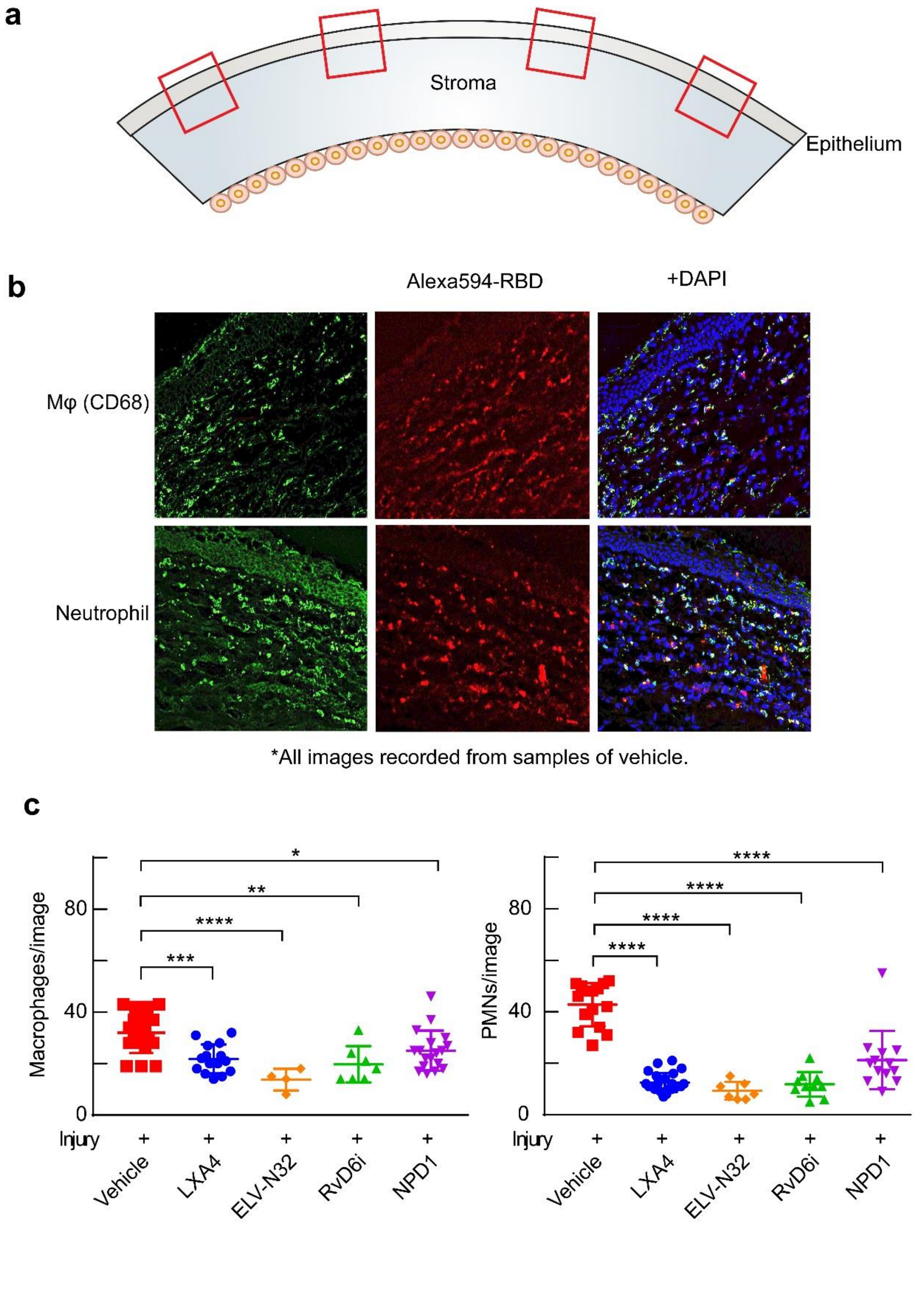
a
Stroma
Epithelium
b
Alexa594-RBD
+DAPI
Mφ (CD68)
Neutrophil
*All images recorded from samples of vehicle.
c
Macrophages/image
PMNs/image
80
40
0
*
**
***
****
Injury
+
Vehicle
LXA4
ELV-N32
RvD6i
NPD1

Supplementary Figure 2. a, Illustration of unbiased microscopy analysis of the rat cornea. Four images were taken (red boxes) for cornea sections. **b**, Representative images stained with CD68 and Neutrophil antibodies and with Alexa 594-RBD of a rat injured cornea. DAPI used to label the nuclei. **c,** Quantification of macrophage (+CD68 cells) and neutrophil. Each data point represents number of cells /cross-section image. Values are means ±SD and p-values calculated by ANOVA-post hoc Dunnett's multiple comparisons test with vehicle as reference. *, $p < 0.05$, **, $p < 0.01$, ***, $p < 0.001$, and ****, $p < 0.0001$.

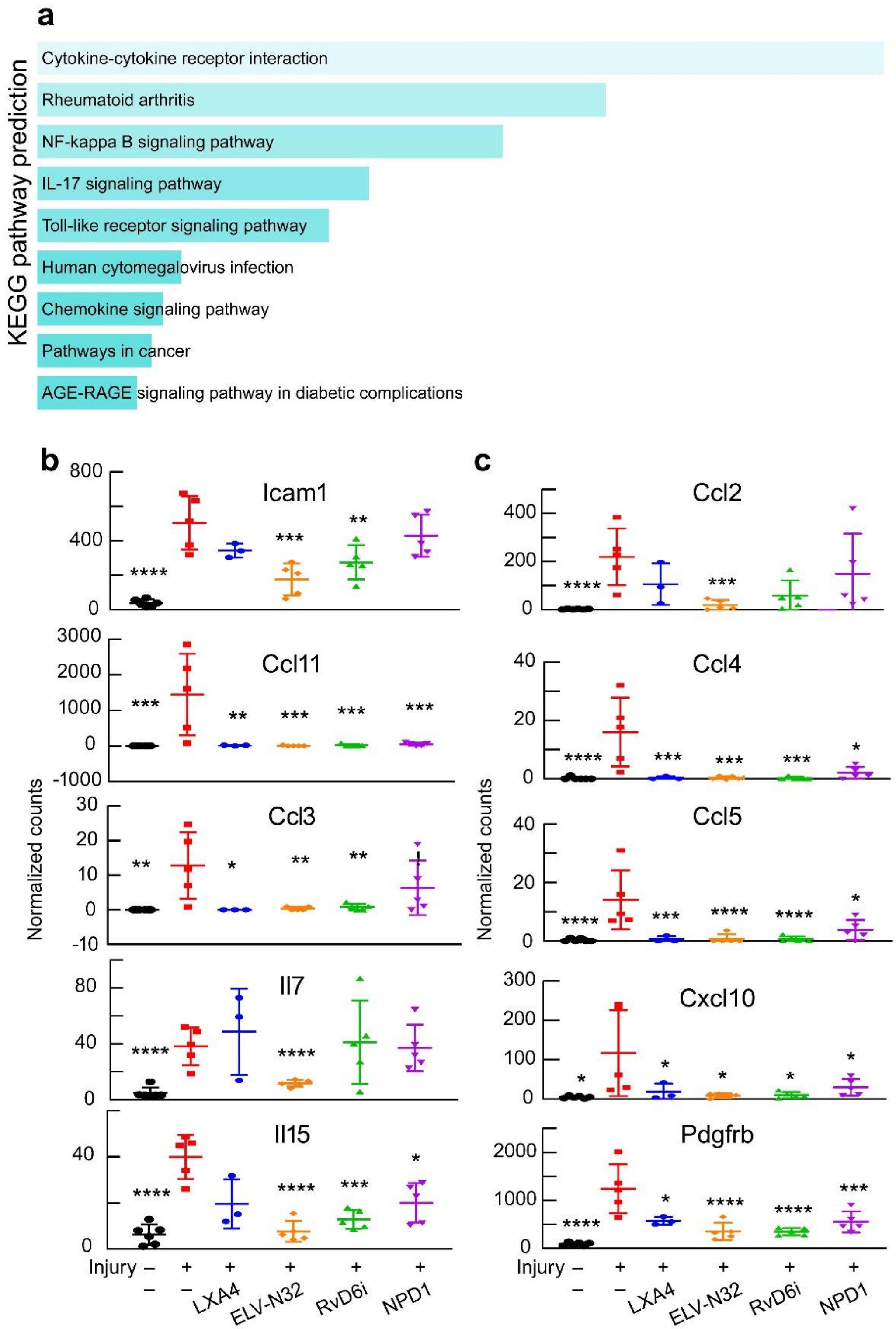
a
KEGG pathway prediction
Cytokine-cytokine receptor interaction
Rheumatoid arthritis
NF-kappa B signaling pathway
IL-17 signaling pathway
Toll-like receptor signaling pathway
Human cytomegalovirus infection
Chemokine signaling pathway
Pathways in cancer
AGE-RAGE signaling pathway in diabetic complications
b
Icam1
Ccl11
Ccl3
Il7
Il15
Normalized counts
c
Ccl2
Ccl4
Ccl5
Cxcl10
Pdgfrb
Normalized counts
Injury – + + + + +
– – LXA4 ELV-N32 RvD6i NPD1
Injury – + + + + +
– – LXA4 ELV-N32 RvD6i NPD1

Supplementary Figure 3. Lipid mediators attenuate expression of cytokine-storm related genes and SASP after cornea injury. **a**, KEGG-pathway enrichment of 51 genes depicted in Fig. 3**b**. Bars were sorted by p-value. The length of the bar represents the significance of the pathway, while the lighter the color, the more significant. The number shows the amount of genes from the denoted group that are enriched in each pathway. **b**, RNA-seq gene expression share between cytokine-storm markers and SASP. **c**, RNA-seq gene expression of cytokine storm-related genes. Normalized counts, (mean and SD) analyzed by ANOVA-post hoc Dunnett's multiple comparisons test with vehicle as reference. *, $p < 0.05$, **, $p < 0.01$, ***, $p < 0.001$, and ****, $p < 0.0001$.

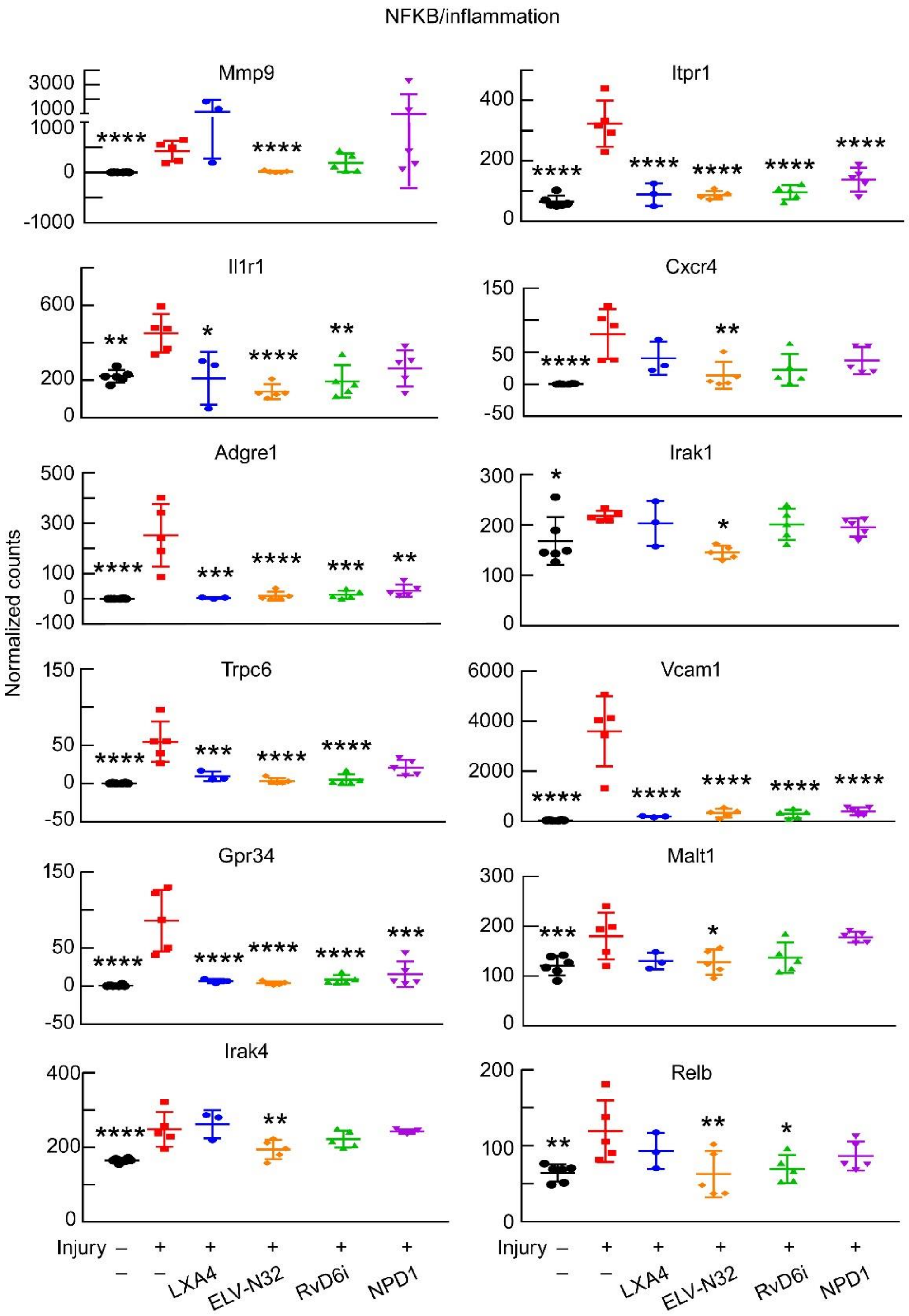
NFKB/inflammation
Normalized counts
Mmp9
Itpr1
Il1r1
Cxcr4
Adgre1
Irak1
Trpc6
Vcam1
Gpr34
Malt1
Irak4
Relb
Injury – + + + + +
– – LXA4 ELV-N32 RvD6i NPD1

Supplementary Figure 4. Effect of lipid mediators on expression of NFkB inflammatory genes after cornea injury. Normalized counts, (mean and SD) analyzed by ANOVA-post hoc Dunnett's multiple comparisons test with vehicle as reference *, $p < 0.05$, **, $p < 0.01$, ***, $p < 0.001$, and ****, $p < 0.0001$.

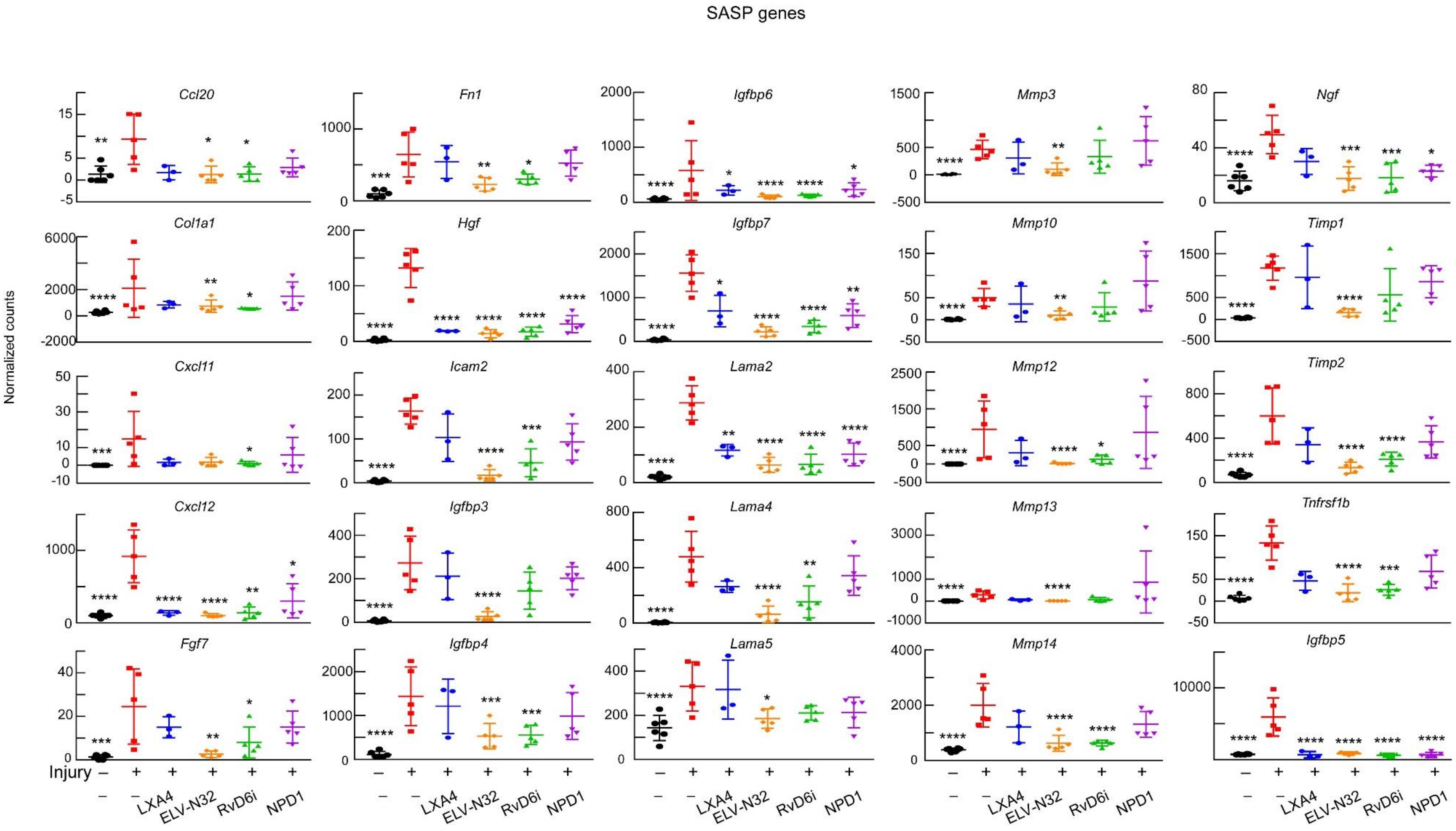

Supplementary Figure 5. Differential effect of lipid meditators on senescence programming gene expression after cornea injury, RNA-seq gene expression. Normalized counts, (mean and SD) analyzed by ANOVA-post hoc Dunnett's multiple comparisons test with vehicle as reference. *, p < 0.05, **, p < 0.01, ***, p < 0.001, and ****, p < 0.0001.

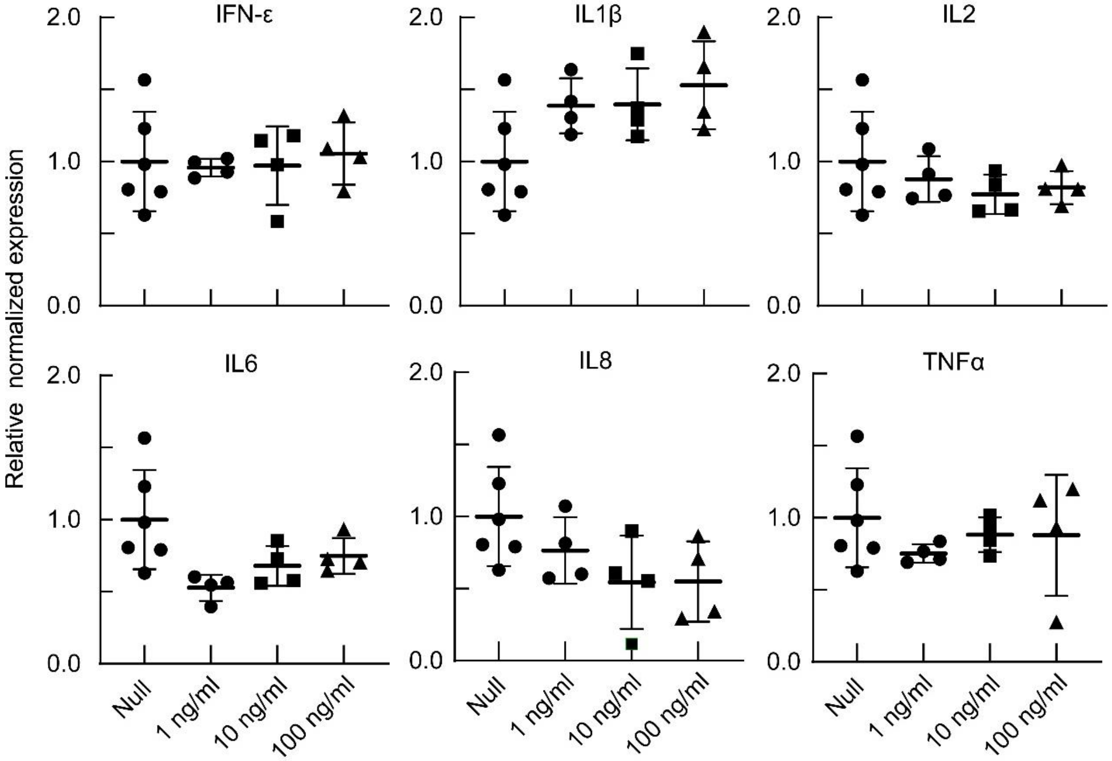

Supplementary Figure 6. dd-PCR gene expression analysis of *Ace2* in HCEC after stimulation with 1, 10, and 100 ng/mL IFNε, IL1β, IL2, IL6, IL8, and TNFα. There was no significant increase with any of the cytokines.

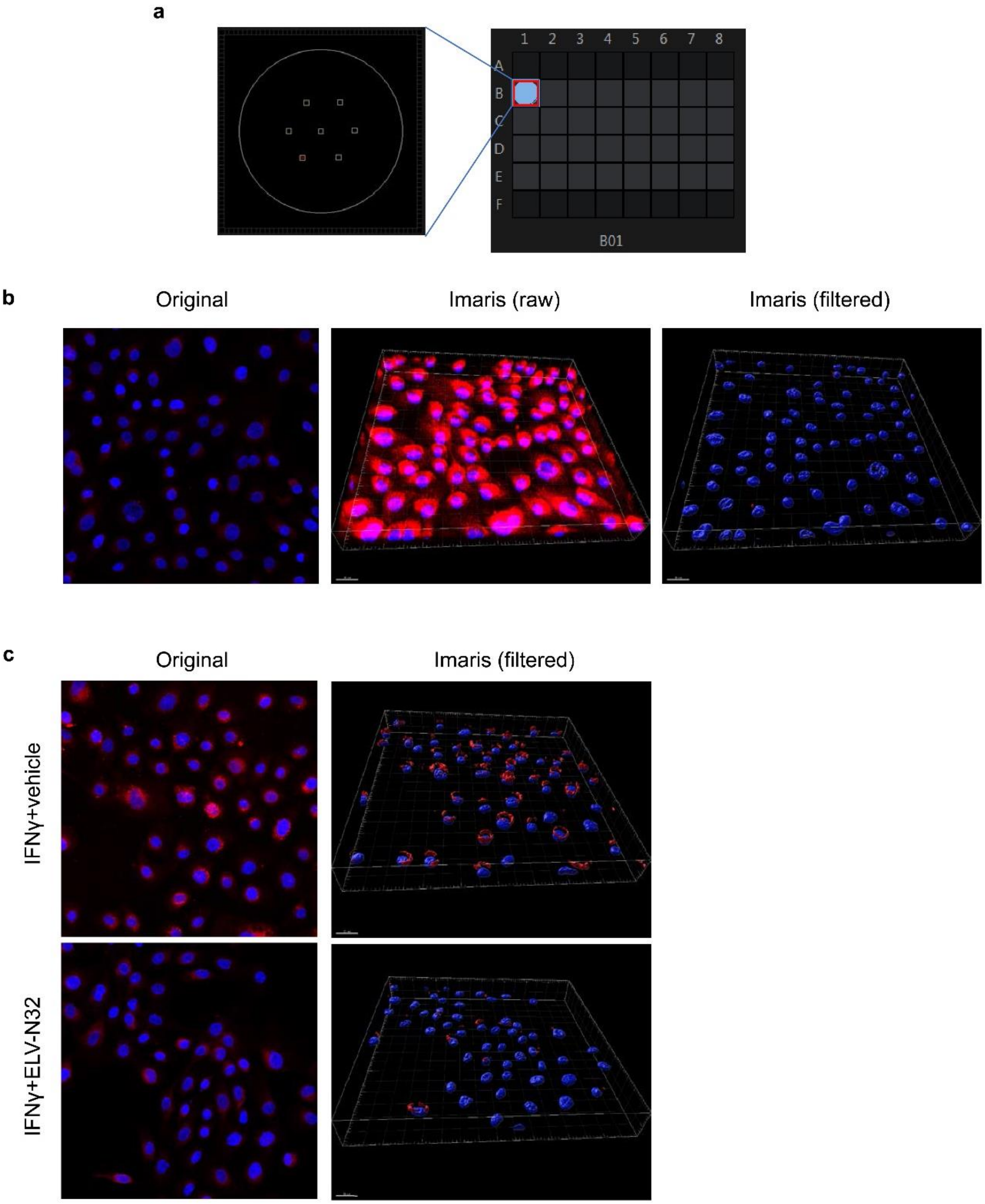

Supplementary Figure 7. a-b. Unbiased imaging analysis for RBD binding in HCEC. **a**, Images were taken in the Multi Area Time Lapse mode with an Olympus FV3000 confocal microscope. For each well, 7 designed areas were taken with the same parameters and Z-section range. **b**, Images of a normal cornea showing the Imaris auto-fluorescence and the filtered image. All images were converted and inputted in

the Imaris software, and the threshold for the control images (HCEC without Alexa 594-RBD) was defined. Then, the batch image processing was used to analyze all images with the defined threshold. The total sum intensity for each image was employed to evaluate binding efficiency. **c,** Representative images of Alexa 594-RBD for vehicle and ELV-N32 treated HCEC from the microscopy (left) and after Imaris threshold-filtration (right).
